# Surveillance, epidemiology, and impact of the coronavirus disease 2019 interventions on the incidence of enterovirus infections in Nanchang, China, 2010–2022

**DOI:** 10.3389/fmicb.2023.1251683

**Published:** 2023-10-18

**Authors:** Xianfeng Zhou, Ke Qian, Chunlong Zhu, Liu Yi, Junling Tu, Shu Yang, Yanxia Zhang, Yanglin Zhang, Wen Xia, Xiansheng Ni, Tielong Xu, Fenglan He, Hui Li

**Affiliations:** ^1^Cancer Research Center, Jiangxi University of Chinese Medicine, Nanchang, China; ^2^Jiangxi Provincial Health Commission Key Laboratory of Pathogenic Diagnosis and Genomics of Emerging Infectious Diseases, Nanchang Center for Disease Control and Prevention, Nanchang, China; ^3^Clinical Laboratory, Third Hospital of Nanchang, Nanchang, China; ^4^School of Life Science, Jiangxi University of Chinese Medicine, Nanchang, China

**Keywords:** enteroviruses, HFMD, phylogenetic analysis, seasonality, COVID-19, ARIMA

## Abstract

**Introduction:**

Pathogen spectrum of Hand, foot and mouth disease (HFMD) has substantially changed in the past decade in China. Growing evidence has indicated that anti-COVID-19 nonpharmaceutical interventions (NPIs) can support control of various infectious diseases, including intestinal diseases.

**Methods:**

In this study, HFMD cases were enrolled from sentinel hospitals of Nanchang, Jiangxi province, and enteroviruses were genotyped using specific real time RT-PCR. We systematically characterized the epidemiology of HFMD based on the continuous molecular surveillance and estimated the impact of COVID-19 intervention on HFMD incidence using seasonal autoregressive integrated moving average (ARIMA) models.

**Results:**

A total of 10247 HFMD cases were included during 2010-2022, of which 6121 enterovirus (EV)-positive cases (59.7%) were identified by real-time RT-PCR. Over 80% cases were associated with EV-A71 and coxsackievirus A16 (CVA16) during 2010-2012, while the type distribution significantly changed as CVA6 emerged to be dominant, accounting for 22.6%-59.6% during 2013-2022. It was observed that the prevalence patterns of EV-A71 and CVA16 were similar and both of them peaked in the second quarter and then leveled off. However, CVA6 was generally prevalent around the fourth quarter, demonstrating a staggered prevalence during 2010-2019. During the COVID-19 epidemic, the seasonal HFMD epidemic peak was restrained, and the ARIMA analysis indicated that the COVID-19 intervention had mitigated EV transmission during the first COVID-19 outbreak in early 2020. In addition, bivariate Spearman’s cross-correlation coefficients were estimated for the major types CVA6, CVA16 and EV-A71. Our analyses indicated the possible existence of correlations among CVA6, CVA16 and EV-A71 prevalence in the epidemiological level.

**Discussion:**

Taken together, the type distribution of HFMD has substantially changed over the last decade and CVA6 and CVA16 are currently the most predominant types co-circulating in Nanchang. The anti-COVID-19 NPIs significantly reduced the incidence of EV infections.

## Introduction

Hand, foot, and mouth disease (HFMD) is a highly contagious disease in children caused by several human enteroviruses (EV) (Xing et al., 2014; Zhou et al., 2015; Zhu et al., 2023). Enteroviruses belong to the family *Picornaviridae*, genus *Enterovirus*. Since 1999, human EVs have been divided into four species of EVs: EV-A (25 serotypes), EV-B (63 serotypes), EV-C (23 serotypes), and EV-D (5 serotypes) based on their biological and genetic characteristics (Solomon et al., 2010). There are various pathogens associated with HFMD outbreaks, particularly EV-A71 and coxsackievirus A16 (CVA16) prior to 2012. Since then, CVA6 and CVA10 are responsible for a significant proportion of HFMD cases and outbreak (Xie et al., 2020). EVs possess similar positive single-strand RNA genome (~7,500 nucleotides) composed of a large open reading frame (ORF) flanked by 5′ and 3′ untranslated regions (UTRs). The 5′ part of the ORF encodes the structural proteins that form the capsid, while the 3′ part of the ORF encodes the non-structural proteins (Palacios and Oberste, 2005). Children under the age of 5 are primarily by HFMD but HFMD can also infect teenagers and adults (Xing et al., 2014; Zhou et al., 2015). Clinical manifestations of HFMD include mild to severe rash, herpangina, pulmonary edema, circulatory disturbances, meningitis, aseptic encephalitis, and even death (Wang et al., 1999; Solomon et al., 2010). Since 2012, outbreaks associated with CVA6 have been frequently reported in Europe, Japan and some developed regions of China (Feng et al., 2015; Kanbayashi et al., 2017; Kamau et al., 2021; Liu et al., 2021; Tomba Ngangas et al., 2022). From then on, CVA6 has been gradually predominant in most areas of China since 2013 (He et al., 2021; Liu et al., 2021). Our former study found CVA6 increasingly predominated in local HFMD cases, and EV-A71 was no longer detected from HFMD surveillance after 2 years EV-A71 vaccine promotion and implementation (He et al., 2021).

In early 2020, non-pharmaceutical interventions (NPIs) were implemented in China to reduce and contain the coronavirus disease 2019 (COVID-19) transmission. A national-scale investigation found that these NPIs have substantially reduced the incidence of HFMD in the first wave of COVID-19 (Zhao et al., 2022). Since the first COVID-19 outbreak in most cities of China, NPIs, vaccines and dynamic Zero-COVID policy were implemented to combat SARS-CoV-2 around mainland China till the end of 2022 (Yang and Yu, 2023). However, the impact of these measures on epidemiological and etiologic characteristics of HFMD has not been well-studied. In this study, we retrospectively analyzed the epidemiological and etiological characteristics of HFMD in the southeastern capital city of Nanchang before (2010–2019) and during the COVID-19 pandemic (2020–2022), and estimated the impact of COVID-19 intervention on HFMD incidence. In Nanchang, the HFMD epidemic underwent three different periods from 2010–2022: (1) EV-A71-dominant period from 2010 to 2012; (2) CVA6 emerged to be one of the dominant strains from 2013 to 2016; (3) CVA6 became predominant with the availability and popularization of EV-A71 vaccines since mid-2016. We used the seasonal autoregressive integrated moving average (ARIMA) models and observed that COVID-19 intervention had a substantial impact on local HFMD incidence. Taken together, these findings will help improve disease forecasting and evaluation of HFMD control interventions in the future.

## Materials and methods

### The aim and design of this study

This study aims to characterize the epidemiology and pathogen spectrum change of HFMD in the past 13 years (2010–2022) in Nanchang, China. Based on a continuous sentinel hospital-based HFMD surveillance, this study also aims to explore the impact of COVID-19 interventions on HFMD incidence using seasonal ARIMA models, and to analyze the phylogenetic characteristics of the dominant types. This study was designed on the premise of constant HFMD surveillance and COVID-19 interventions on HFMD incidence with ARIMA models.

### Data collection

**Figure 1:**
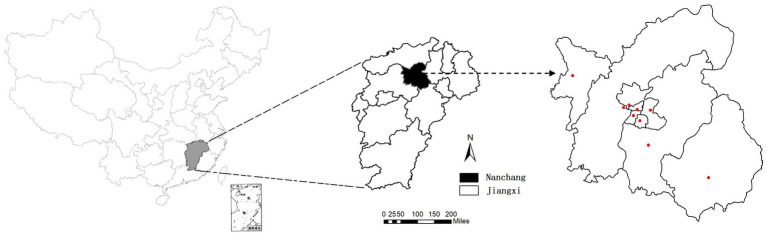
Geographical location of Nanchang City, China. **(A)** Map of China; **(B)** map of Jiangxi, and its capital city Nanchang was marked; **(C)** red dots represent the locations of 9 sentinel hospitals in Nanchang.

Nanchang is a city located at 115°27′–116°35′ E longitude and 28°10′–29°11’ N latitude (Figure 1). Nanchang had a population of 6.4 million as of 2022, accounting for 12.5% of the population of Jiangxi Province. Since 2009, it is mandated that clinical specimens be collected from all severe HFMD cases, and the first 5 mild cases reported per month in each county or district are tested for enteroviruses using real time RT-PCR by the local CDCs according to the HFMD surveillance protocol as previously described (Xing et al., 2014; Li et al., 2018; He et al., 2021).

During 2010–2022, a total of 10,247 clinically diagnosed HFMD cases were collected from 9 hospitals in Nanchang, including Jiangxi Provincial Children’s Hospital and 8 county and district sentinel hospitals. As two or three types of samples were collected from some cases, a total of 11,196 clinical specimens (10,137 pharyngeal/throat swabs, 781 anal swabs and 278 stools) were collected. The laboratory-confirmed cases were determined by real time RT-PCR result of any kind of their samples collected. In this study, a total of 10,247 possible cases were included for the test and analysis.

The study was approved by the ethics committee of the Nanchang Center for Disease Control and Prevention, and the procedures were performed according to the approved guidelines (Approval No. NCCDC-20100701). Prior informed consent was obtained from patients or their parents for sample collection.

### Type identification of enteroviruses

Swabs were stored in a dedicated Universal Transport Medium (UTM) (Yocon, Beijing, China) for transport. Stool samples were diluted to a 10% suspension using MEMs. After thorough mixing, 200 μL of clinical samples were used to extract RNA using the QIAamp Viral RNA Mini Kit (Qiagen, CA) according to the manufacturer’s instructions. Viruses EV-A71, CVA16, CVA6, and CVA10 were confirmed using per commercial real-time RT-PCR Kits with item No. YJC20101, YJC20102, YJC20103, YJC20106, and YJC20107, respectively (BioPerfectus technologies, Jiangsu, China). Samples positive for universal EV but negative for the above types were named as un-typed EV (UEV).

### NPIs data collection during COVID-19 epidemic

Local COVID-19 outbreaks were updated by official websites of Nanchang Municipal Health Commission: http://hc.nc.gov.cn/ncwjw/index.shtml. The time span of each outbreak and level of public health emergency response (PHER) were determined according to the official announcement. According to the documents issued by the local governments based on the Joint Prevention and Control Mechanism about COVID-19, the start and end dates of the lockdown of districts and other public places were collected.

### ARIMA modeling and statistical analysis

Since HFMD has strong seasonality, a seasonal ARIMA model (p,d,q) (P,D,Q) s was used for modeling, where p and P are autoregressive order and seasonal autoregressive order respectively, and q and Q are the moving average and seasonal moving average, respectively. d and D are the difference order and seasonal difference order respectively, and s is the seasonal period. Based on the monthly onset number of HFMD cases, we fit ARIMA models for the pre-COVID-19 period (2010–2019) and used these models to predict the prevalence of HFMD in 2020. Seasonal ARIMA models were fitted and utilized based on following procedures: (a) Sequence stability: time series diagrams from the 1st month of 2010 to the 12th month of 2019 were generated. The sequences of the monthly onset of HFMD cases were transformed into stable sequences; (b) Model recognition and parameter estimation: the order was determined through the time series of the autocorrelation function (ACF) and partial autocorrelation function (PACF); (c) Model diagnostics: the optimal model was determined based on the Akaike information criterion (AIC) value; and (d) Model prediction: the constructed seasonal ARIMA model was used to predict the number of monthly HFMD cases in Nanchang in 2020 (months 1–12), assuming that there was no COVID-19-associated NPIs (Zhao et al., 2022). This analysis was done by ARIMA Forecasting (v1.0.11) in Free Statistics Software (v1.2.1), Office for Research Development and Education, URL http://www.wessa.net/rwasp_arimaforecasting.wasp/.

Spearman’s rank correlation coefficients were computed and tested between all pairs of virus infection prevalence (the proportion positive among those tested) in each month using GraphPad Prism 8. We additionally conducted an analysis of the distribution of correlation coefficients generated under the null hypothesis of correlations. To do so, we randomly permuted the monthly prevalence time series of each virus pair 1,000 times and computed the 2.5 and 97.5% quantiles of each distribution of correlation coefficients. See Supplementary Tables S1, S2 for the estimated correlation coefficients, distributions under the null hypothesis, and *p* values, respectively.

## Results

### Epidemiology and etiology of HFMD in Nanchang from 2010 to 2022

**Figure 2:**
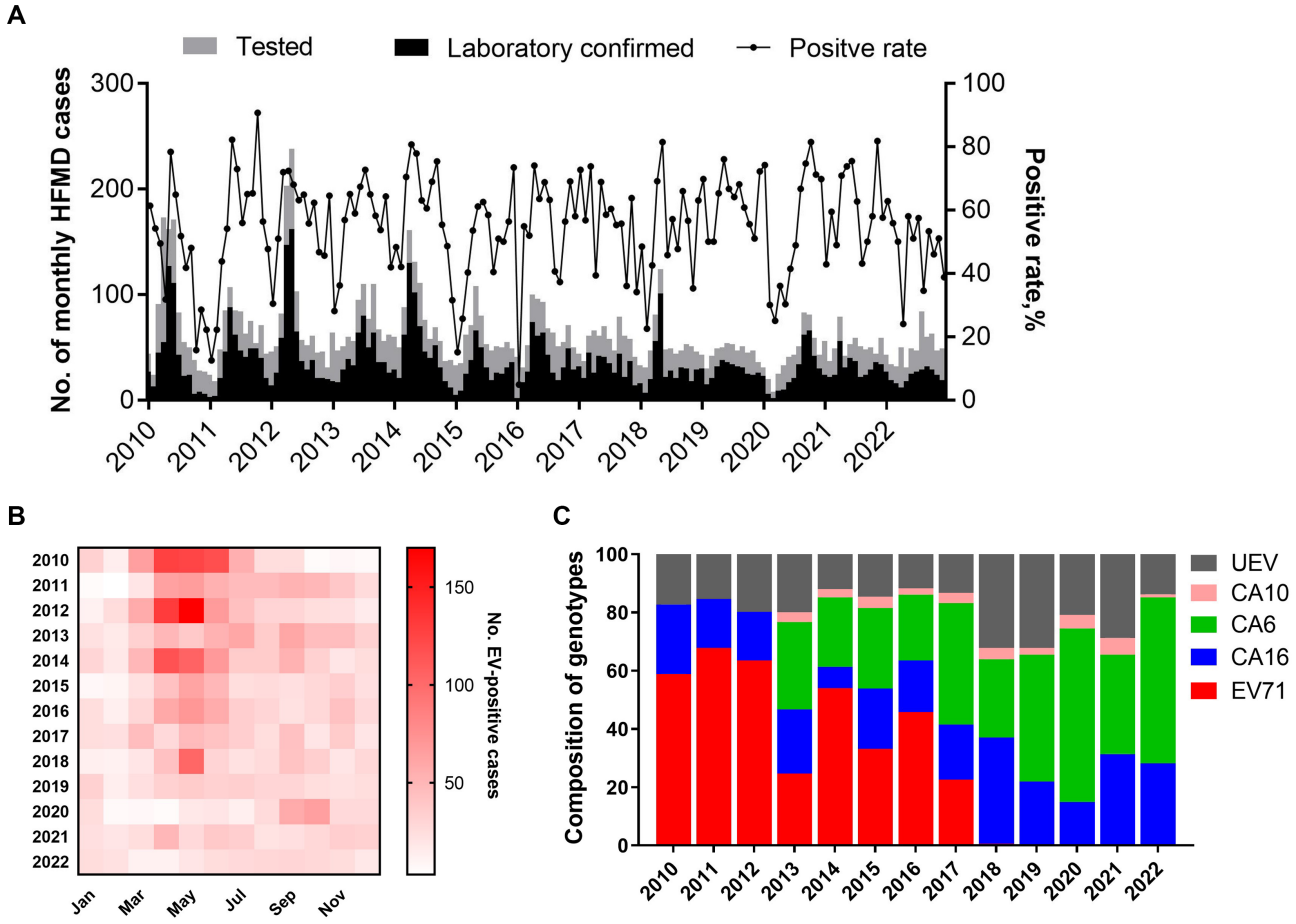
Pathogen spectrum of HFMD in Nanchang during 2010–2022. **(A)** Monthly distribution of HFMD cases and positive rate of EVs from 2010 to 2022; **(B)** heatmap of monthly distribution of laboratory-confirmed HFMD cases in Nanchang from 2010 to 2022. **(C)** Yearly proportion of enterovirus types during 2010–2022 in Nanchang, China (CVA6 and CVA10 were added into surveillance list of pathogens since 2013).

**Figure 3:**
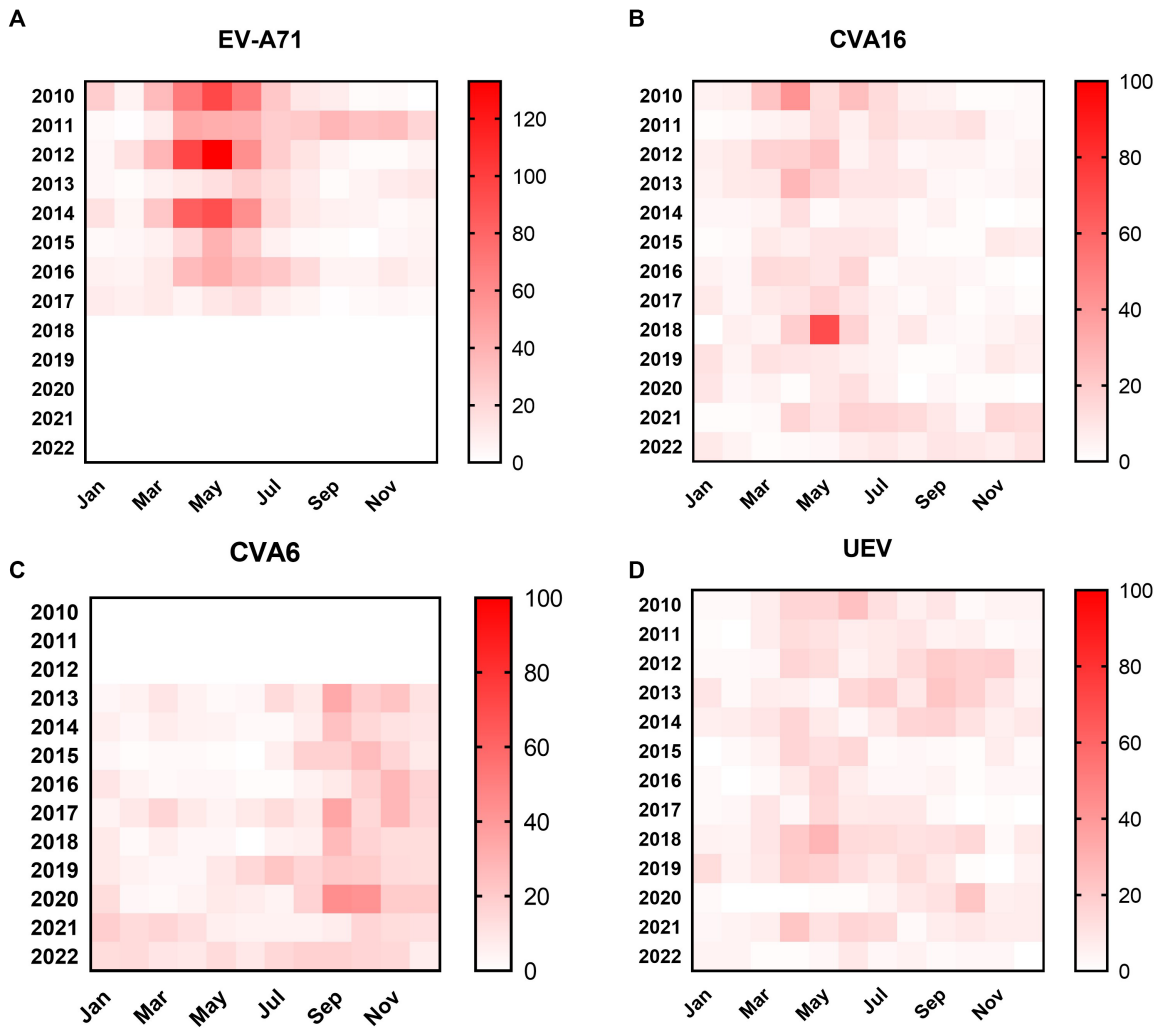
Heatmaps of monthly distribution of EV-positive cases. **(A)** EV-A71-associated cases from 2010–2022; **(B)** CVA16-associated cases from 2010 to 2022; **(C)** CVA6-associated cases from 2010–2022; **(D)** UEV-associated cases from 2010 to 2022.

From January 2010 to December 2022, a total of 10,247 suspected HFMD cases were collected from sentinel hospitals for EV screening in Nanchang City. Among the HFMD cases, 6,121 EV-positive cases (59.7%) were laboratory confirmed by real-time RT-PCR. It was found that the positive rate of EV positively correlated with the number of tested HFMD cases (*r* = 0.452, *p* < 0.0001) as Figure 2A indicated. Years 2010, 2012, and 2014 had the highest number of tested HFMD cases (Figures 2A,B). Our previous studies found that subtype C4a of EV-A71 was the predominant causative agent of HFMD in children in Nanchang (Zhou et al., 2015). Since 2017, the HFMD epidemic has been generally stable, fluctuating in a lower prevalence (Figure 2A). Generally, the incidence of HFMD peaked in the second quarter (Q2) during 2010–2018 (Figures 2A,B). As surveillance data indicated, changes in type distribution of HFMD can be roughly divided into three periods in Nanchang (Figure 2C). Firstly, over 80% cases were infected with EV-A71 (>60%) and CVA16 (~20%) during 2010–2012. Secondly, the pathogen spectrum significantly changed as CVA6 emerged to be one of the dominant types, accounting for 22.6–41.7% during 2013–2017, in which the EV-A71 vaccines were available since June 2016 and caused a significant drop of EV-A71 infections by 2017 (Figure 2C). Thereafter, with rising rates of EV-A71 vaccination, EV-A71 has not been detected from HFMD cases since 2018, followed by two predominant pathogens CVA6 and CVA16 alternately co-circulating in Nanchang (Figure 2C). More specifically, the type distribution of HFMD had a substantial change after the launch of EV-A71 vaccination in mid-2016, the typical biennial outbreak pattern gradually developed into less volatile mode as EV-A71-associated cases substantially decreased (Figures 2B, 3A). For each type, it was observed that the prevalence patterns of EV-A71 and CVA16 were similar and both of them peaked in Q2 and then leveled off (Figures 3A,B). However, CVA6 was generally prevalent around Q4 (Figure 3C). As for other un-typed EVs (UEV), there was no typical seasonality as surveillance data indicated (Figure 3D).

### Age and gender distributions of hand, foot, and mouth disease

**Figure 4:**
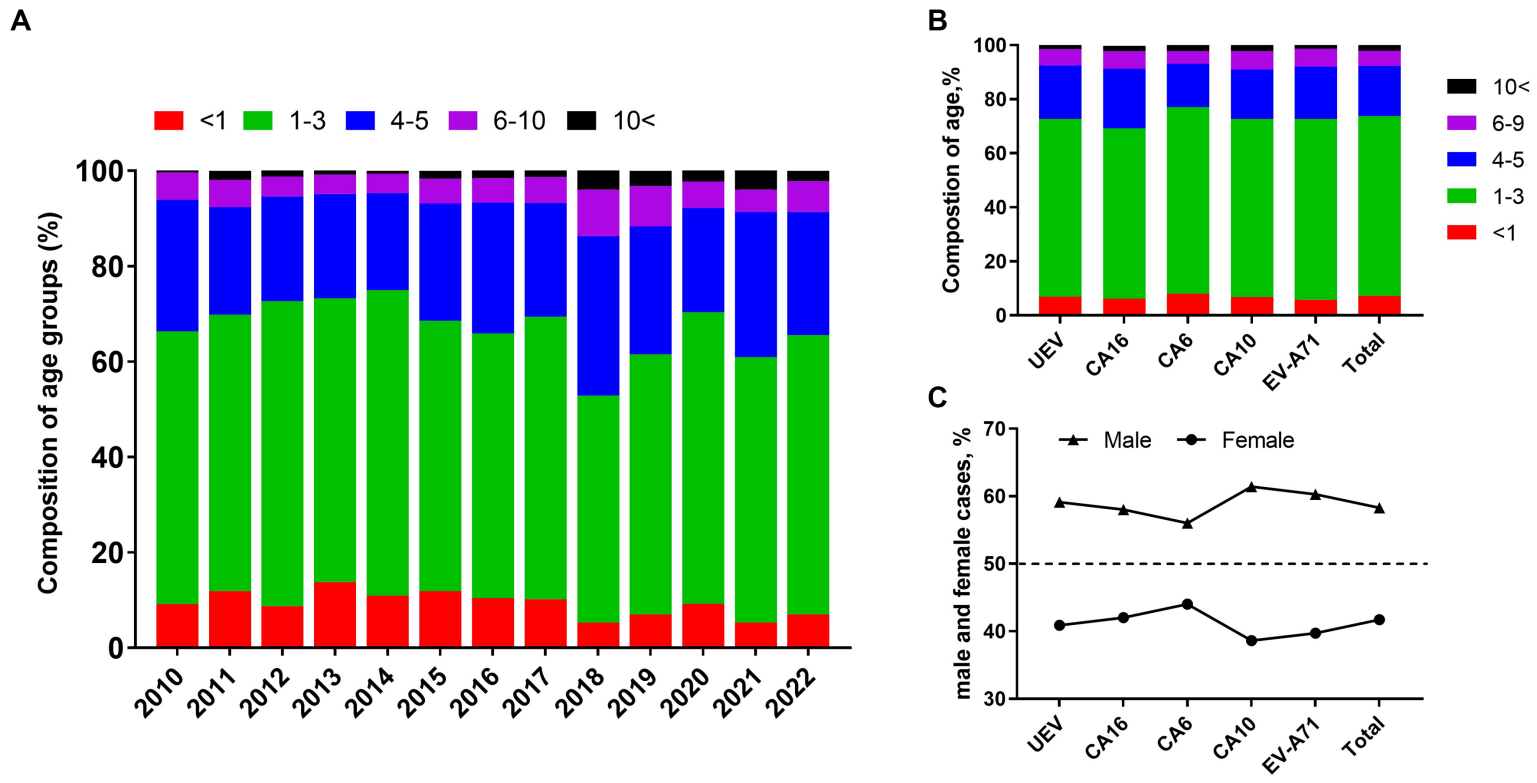
Characteristics of gender distribution and composition of age groups in HFMD cases. **(A)** Composition of age groups from 2010 to 2022; **(B)** composition of age groups in different types of enteroviruses; **(C)** proportion of male and female cases in different types of enteroviruses.

Generally, pre-school children are the highest risk population of HFMD. In this study, we found children aged 1–3 years accounted for 57.78% (95% CI: 55.20–60.36%) of laboratory-confirmed cases, with an average of 9.29% (95% CI: 7.71–10.87%) of children under 1 year of age. Children aged 4–5 years accounted for 25.26% (95% CI: 22.96–27.56%), while only 5.74% (95% CI: 4.73–6.76%) of cases occurred in children aged 6–9 years. Infections in people aged 10 years and older were rare, accounting for approximately 1.93% (95% CI, 1.22–2.64%) (Figure 4A). The results indicated that HFMD generally affects pre-school children and children under 3 years of age were at the highest risk of enterovirus infection. For each type, there was no significant difference of age composition (Figure 4B). To explore gender differences, we found male cases were 1.39 times more common than females, and the male-to-female ratios of cases associated with CVA6, CVA10, CVA16, and EV-A71 were 1.30, 1.59, 1.40, and 1.43, respectively (Figure 4C).

### The impact of COVID-19 intervention on the incidence of EV infections in Nanchang

**Figure 5:**
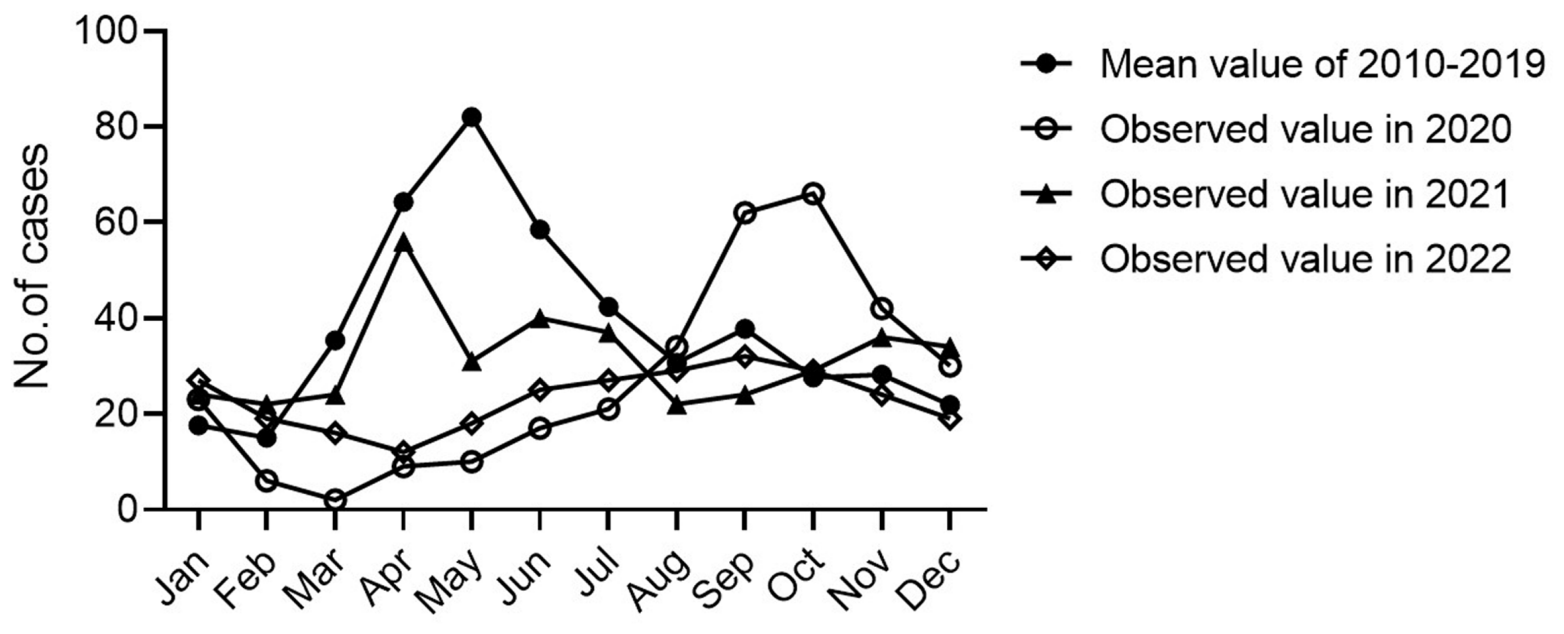
Monthly time-series of HFMD cases in Nanchang, 2010–2022.

Since January 25, 2020, the first COVID-19 cases were reported in Nanchang, leading to an outbreak of 230 SARS-CoV-2-associated cases from January 25 to March 11, 2020. During this period, the strict NPIs along with the first-level PHER was implemented. By comparing the differences in seasonal HFMD incidence in the time series for 2020, 2021, 2022 and the previous years, we found that in contrast to the characteristic trends for the average number of HFMD cases from 2010 to 2019, the primary characteristic peak disappeared in 2020 corresponding to the emergency response concurrently performed by the government (Figure 5). Thereafter, the trend returned to a relatively lower level in 2021 exhibiting a similar trend to the variations in 2010–2019.

**Figure 6:**
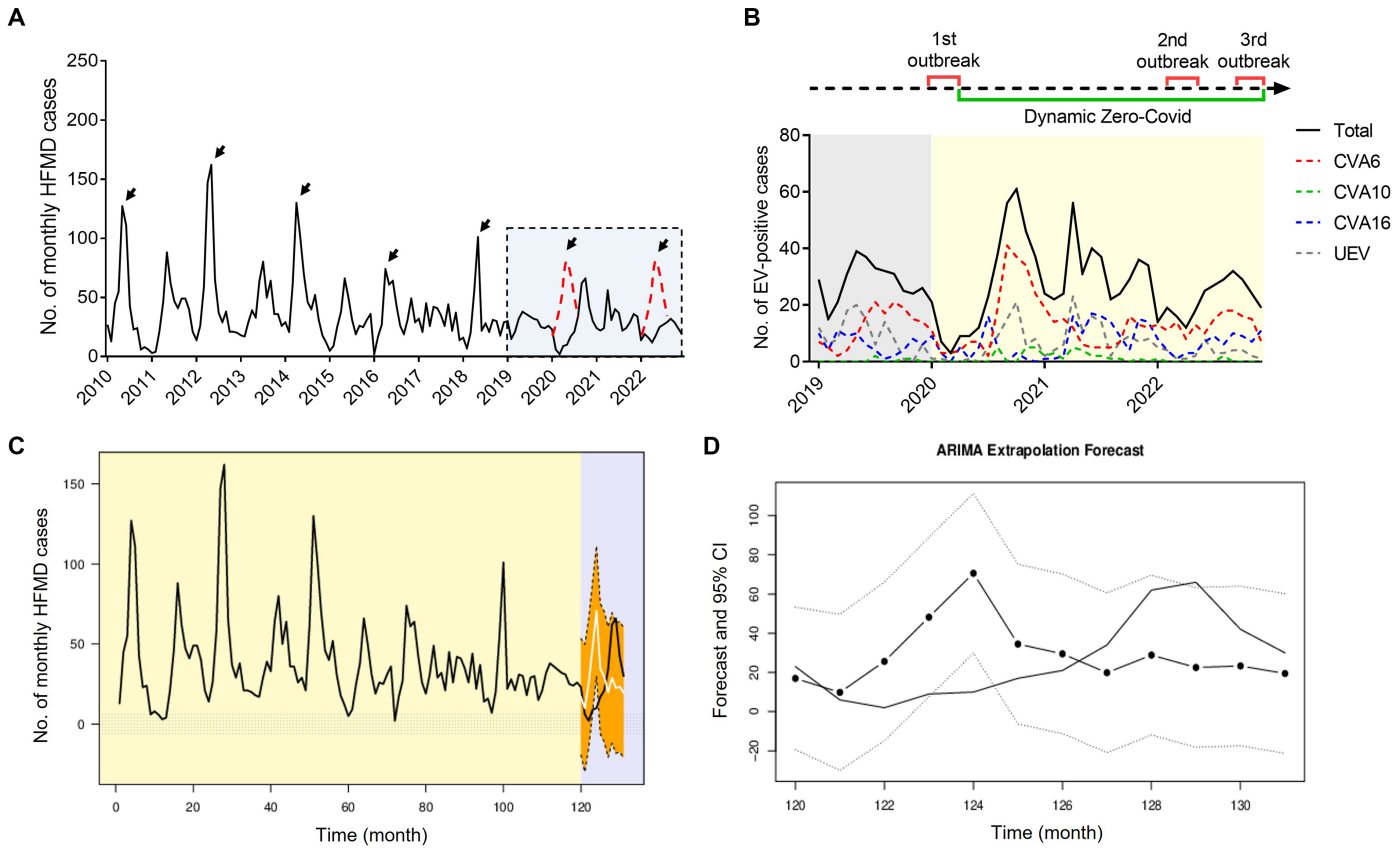
Impact of COVID-19 interventions on HFMD incidence in Nanchang using ARIMA models. **(A)** Monthly curve of EV-positive HFMD cases, black arrows indicate biennial peaks around May from 2010 to 2022, red dotted peaks are inserted as possible presumption to follow the seasonality pattern; **(B)** time line of three waves of local COVID-19 outbreaks and monthly curve of CVA6-, CVA10-, CVA16-, and UEV-infected cases in Nanchang from 2019 to 2022. 1st outbreak: January 25to March 11, 2020; 2nd outbreak: March 16 to May 5, 2022;3rd outbreak: December 2022; **(C)** observed HFMD case counts in Nanchang from 2010 to 2020, compared with the fitted (2010–2019) and predicted (2020) case counts obtained using the ARIMA models in the absence of COVID-19 outbreaks. The light-purple shaded part indicates the observed (black) and estimated (white) case counts from early January to the end of December 2020; **(D)** zoom-in view of the estimated case counts of 2020. The dotted line indicates 95% CI of estimated cases.

Since the establishment of HFMD surveillance, we observed a typical biennial outbreak pattern of HFMD peaking around May of the even-numbered years from 2010 to 2019 (Figure 6A). However, the peaks did not occur around May of 2020 and 2022 as expected (Figure 6A). During the first local COVID-19 outbreak, the HFMD incidence plunged and the expected epidemic peak vanished as a result of a city-level lock down along with the first-level PHER (Figure 6B). This extrapolation was strongly supported by ARIMA forecasting that predicted a peak of HMFD cases around May 2020 in the absence of NPIs (Figures 6C,D). From then on, the dynamic Zero-COVID policy continued until December 2022 (Figure 6B) (Yang and Yu, 2023). As shown in the Figures 5 6B, the predicted biennial peak of HFMD incidence did not occur around May 2022 but a low-volatility plateau of HFMD incidence, which might be caused by the constant NPIs during the 2nd outbreak from March 16 to May 6 2022 (Figure 6B).

### Potential correlation of viral prevalence of different types

**Figure 7:**
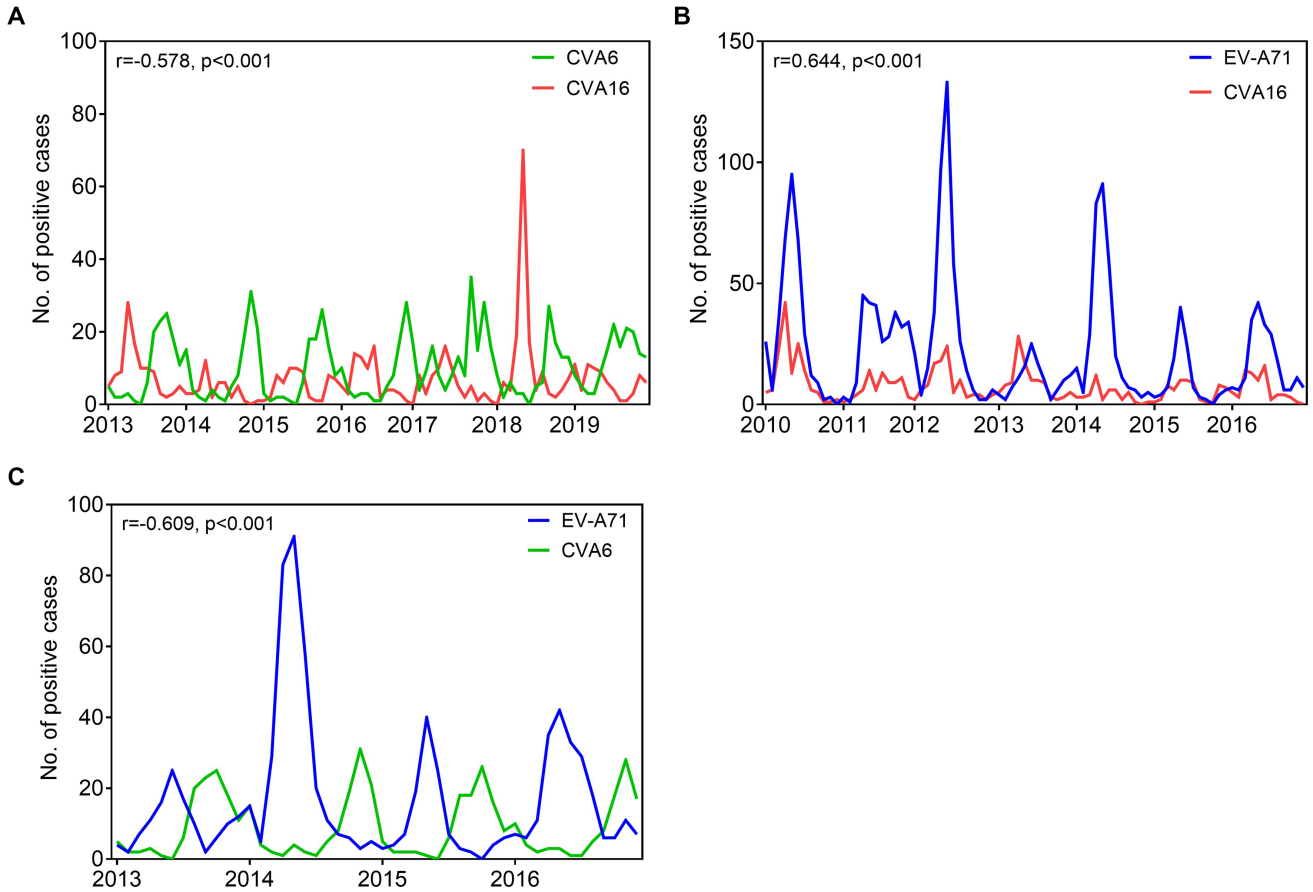
Comparative prevalence of monthly EV infections detected among HFMD cases in Nanchang, China. **(A)** Comparative prevalence of CVA6 and CVA16 from 2013 to 2019; **(B)** comparative prevalence of EV-A71 and CVA16 from 2010 to 2016; **(C)** comparative prevalence of CVA6 and EV-A71 from 2013 to 2016. Asynchronous seasonality, explained by negative epidemiological correlations. Synchronous seasonality, explained by positive epidemiological correlations. *r*, correlation coefficients; *p*, *p* value.

In Nanchang, CVA6 and CVA16 are the major causative agents circulating in Nanchang, while these two viruses demonstrated a staggered prevalence pattern during 2013–2019 as shown in Figure 7A. To explore the potential correlations of viral infections of different types, we conducted a pair correlation analysis for CVA16, CVA6, and EV-A71 prevalence before the COVID-19 epidemic. As expected, CVA6 and CVA6 prevalence negatively correlated (*r* = −0.578, *p* < 0.001) during 2013–2019 (Figure 7A). Correlations of CVA6 and CVA16 prevalence versus EV-A71 prevalence during 2010–2016 and 2013–2016 were, respectively, analyzed to rule out the impact of EV-A71 vaccination that indicated since mid-2016. It’s found that EV-A71 and CVA16 prevalence displayed a positive correlation (*r* = 0.644, *p* < 0.001), while EV-A71 and CVA6 prevalence were negatively correlated (*r* = −0.609, *p* < 0.001) (Figures 7B,C and Supplementary Tables S1, S2). The estimated cross-correlations fall outside the 2.5 and 97.5% quantile intervals of correlation distributions generated under the null hypothesis of no correlation (Supplementary Tables S1, S2).

## Discussion

From 2008 to 2012, EV-A71 was the major pathogen causing HFMD and was responsible for most severe and fatal cases in mainland China (Xing et al., 2014). Our previous studies have shown that locally circulating EV-A71 strains belong to the C4a sub-genotype, which is a dominant genotype causing most of severe cases and death in mainland China during 2008–2012 (Chen et al., 2014; Gao et al., 2014; Zhou et al., 2015; Ji et al., 2019). However, the type distribution of HFMD substantially changed as CVA6 increasingly replaced EV-A71 to be predominant in many cities of China, particularly after the launch of EV-A71 vaccination since 2016 (Li et al., 2014). We formerly observed that the proportion of EV-A71 continued to decrease as vaccination rates increased, at 22.5% in 2017, 0.5% in 2018 and disappeared from 2019, suggesting that immune protection in children is quite efficient after programmed EV-A71 vaccination (He et al., 2021). However, the EV-A71 vaccines has no cross protection for other types, leading to a substantial change of pathogen spectrum pattern in China. In this study, we characterized the pathogen spectrum pattern of HFMD based on 13 years surveillance in Nanchang, and analyzed the potential impact of COVID-19 intervention on HFMD incidence using ARIMA model. The seasonality pattern of HFMD was similar with other cities of China in the absence of COVID-19 intervention (Li et al., 2018; Ji et al., 2019). In this study, the peaks of HFMD incidence shifted with the impact of COVID-19 in Nanchang, which was also observed in other Chinese cities during this period (Shen et al., 2022; Zhao et al., 2022).

**Figure 8:**
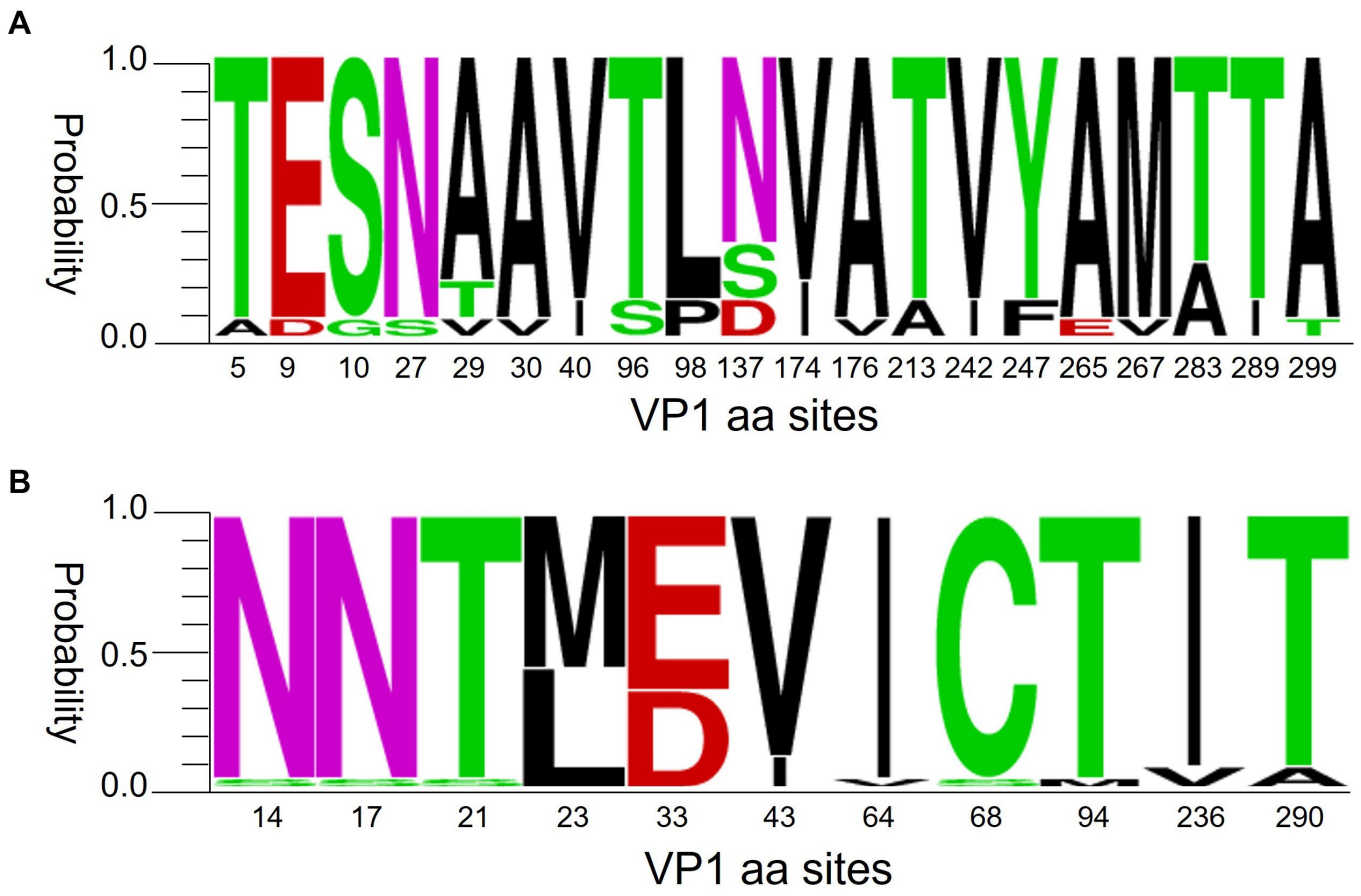
Polymorphism of VP1 amino acids of CVA6 **(A)** and CVA16 **(B)**. The figures were built using weblogo.berkeley.edu/logo.cgi.

The COVID-19 pandemic and subsequent implementation of NPIs (e.g., cessation of global travel, wearing mask, physical distancing, and staying home) reduced transmission of some viral respiratory pathogens, most notably pediatric respiratory syncytial virus (RSV) and influenza (Chow et al., 2023). Here, we evaluated the impact of NPIs of COVID-19 on the transmission of enteroviruses, and found the HFMD incidence substantially decreased during the first outbreak of COVID-19 in early 2020 (Figure 8). The findings supported a previous study which observed a national-scale (covering 31 provincial capitals in mainland China) impact of NPIs on HFMD incidence in 2020 (Zhao et al., 2022). Recently, a report from Xi’an also observed similar findings indicating that NPIs of anti-COVID-19 had a significant impact on HFMD incidence in early 2020 (Shen et al., 2022). This study provides a preliminary effort in revealing how NPIs specific to COVID-19 acted on the EV transmission in Nanchang, which can be extended to other large cities as a reference in synergistic prevention and control of multiple epidemic diseases. And it will be more convincing to use computer simulations to obtain improved understanding of how the epidemiology of viral infections is interlinked, which can help improve disease forecasting and evaluation of HFMD control interventions in the future.

Results of this study indicated the possible presence of correlations among CVA6, CVA16, and EV-A71 prevalence at the epidemiological level by 13 years continuous surveillance of EV types. However, the limit of this analysis is that some potential confounding factors may have interfered the correlation. To our knowledge, EV-A71 and CVA16 share the same entry receptor SCARB2, while KREMEN1 was proven as an entry receptor for most of the coxsackie type A viruses including CVA2-CVA6, CVA10, and CVA12 (Staring et al., 2018; Tang et al., 2022). Whether the correlation among EVs is interlinked with the entry receptors remains unknown.

Children age less than 5 years are still highest-risk population of EV infection regardless of types and pathogen spectrum fluctuation, and male cases were more common than females in all types of EVs. It’s still unclear what is behind the proneness of gender even though this observation is similar with previous conclusions (Xing et al., 2014; Wang et al., 2018; Ji et al., 2019). However, this issue has been focused by the community in recent years (Gay et al., 2021). Formalin-inactivated EV71 vaccines are currently available for children of 6–59 months in China and substantially mitigated EV-A71 transmission (Head and Keynan, 2019; He et al., 2021). The ARIMA models based on HFMD cases and EV-A71 cases effectively present the protective effect of EV-A71 vaccines against EV-71 infections in 2017, leading to lower incidence of HFMD and EV-A71-infection in Nanchang (Supplementary Figures S1A,B). However, these vaccines fail to confer cross-protection against CVA16 (Supplementary Figure S1C), highlighting the necessity of developing a multivalent HFMD vaccine. Although access to EV-A71 vaccine is convenient in Nanchang, we observed a downward trend of EV-A71 vaccination that was likely due to the NPIs (Supplementary Figure S2). However, follow-up of vaccination rate and public health education are necessary to consolidate the achievements of elimination of EV-A71 infection.

Enteroviruses EV-A71, CVA16, and CVA6 are the major EVs that cause HFMD worldwide. There are no standardized criteria for the classification of subtype (Lukashev et al., 2018). Bayesian phylogenetic methods with an integrated molecular clock were introduced a decade ago and provided unprecedented opportunities for phylogenetic analysis. The genetic evolution of EV-A71 virus can be divided into seven genotypes (A–G), with genotypes B and C further divided into sub-genotypes B1–B5 and C1–C5, respectively (Brown et al., 1999). CVA16 is divided into 2 major genogroups A and B with genogroup B being further divided into B1 and B2 (Nhu et al., 2021). Sub-genotype B1 can be further divided into clusters B1a, B1b, and B1c. B1a and B1b can be found in China, Malaysia, Thailand, Australia, and France, which indicate that they evolve and co-circulate all over the world (Hu et al., 2021). Previous studies revealed that CVA6 strains could be divided into 6 genotypes designated as A to F, and D genotypes could be further subdivided into D1-3 sub-genotypes. In recent years, the D genotype, particularly D3 sub-genotype, has become the dominant sub-genotype circulating in Southeast Asia and Europe (Fu et al., 2020; Song et al., 2020; He et al., 2021).

Previous evidence suggests that CVA6 began sporadically spreading in China from late 2012 before turning dominant in 2013 (Feng et al., 2015; Song et al., 2017; Li et al., 2018; Wang et al., 2018). Despite the lack of publicly available CVA6 surveillance data after 2015, our survey observed a dominant trend of CVA6 in Nanchang (Figure 2C). Nevertheless, CVA16 has been sustaining a stable proportion (21.31, 95% CI: 16.74–25.88%) and low-volatility pattern from 2010 to 2022. Our former phylogenetic analysis indicated that D3 CVA6 was the dominant sub-genotype circulating in Nanchang, Jiangxi over the past 10 years (He et al., 2021). Recent studies indicated that B1b CVA16 was the predominant sub-genotype of CVA16 circulating in mainland China (Chen et al., 2019; Hu et al., 2021). According to the polymorphism of VP1 amino acids of representative CVA6 and CVA16 strains of Nanchang, Jiangxi, these two dominant strains had different levels of mutation of VP1 amino acids (Figure 8). However, how do these mutations influence the evolutionary direction of the viruses remains unclear. Therefore, studies on the phylogenetic genomics need to be done to clarify the evolution, selective pressure and phylodynamics of these dominant strains.

## Data availability statement

The data presented in the study are deposited in the GenBank repository, accession number: MW075633-MW075642; OL677506-OL677510; OL688664-OL688754.

## Ethics statement

The studies involving humans were approved by The ethics committee of the Nanchang Center for Disease Control and Prevention. The studies were conducted in accordance with the local legislation and institutional requirements. Written informed consent for participation in this study was provided by the participants’ legal guardians/next of kin.

## Author contributions

XZ and FH conceived and designed the study. XZ, CZ, KQ, JT, LY, and FH performed experiments. XZ, LY, FH, YxZ, WX, XN, and TX collected data. XZ, FH, SY, YgZ, and HL analyzed and interpreted the data. XZ and HL wrote the manuscript. All authors contributed to the article and approved the submitted version.
