# Supplementary material for: Surveillance, epidemiology, and impact of the coronavirus disease 2019 interventions on the incidence of enterovirus infections in Nanchang, China, 2010–2022
Source: Front Microbiol. 2023 Oct 18;14:1251683. doi: 10.3389/fmicb.2023.1251683 (PMC10618362; doi:10.3389/fmicb.2023.1251683)

Supplementary Table 1: **Bivariate Spearman's cross-correlation coefficients estimated for each pair of virus infection time series data (monthly prevalence).** Significance was assessed by asymptotic (t approximation) p-values <0.05 (see **Supplementary Table 2**). Red indicates significant positive coefficients and blue indicates significant negative coefficients. Lower (2.5%) and upper (97.5%) quantiles from Spearman's rank coefficient distributions estimated under the null hypothesis of no virus-virus interactions are given in parentheses. The distribution under the null hypothesis was generated by randomly permuting each pair of virus infection time series data (monthly prevalence) 1000 times.

**EV-A71**			
**CVA6**	-0.609 (-0.201, 0.208)		
**CVA16**	0.644 (-0.227, 0.201)	-0.578 (-0.224, 0.229)	
	**EV-A71**	**CVA6**	**CVA16**

Supplementary Table 2: Bivariate cross-correlation asymptotic (t approximation) p-values estimated by Spearman's rank method between each pair of virus infection time series data (monthly prevalence). Significance was based on p-value <0.05; significant positive correlations are in red, significant negative correlations are in blue.

EV-A71			
CVA6	<0.001		
CVA16	<0.001	<0.001	
	EV-A71	CVA6	CVA16

Supplementary Figure 1

Observed monthly HFMD (A), EV-A71 (B) and CVA16 (C) case counts in Nanchang from 2010 to 2017, compared with the fitted (2010-2015) and predicted (2016-2017) case counts obtained using the ARIMA models in the absence of EV-A71 vaccination. The light-purple shaded part indicates the observed (black) and estimated (white) case counts from early January 2016 to the end of December 2017. The dotted line indicates 95% CI.

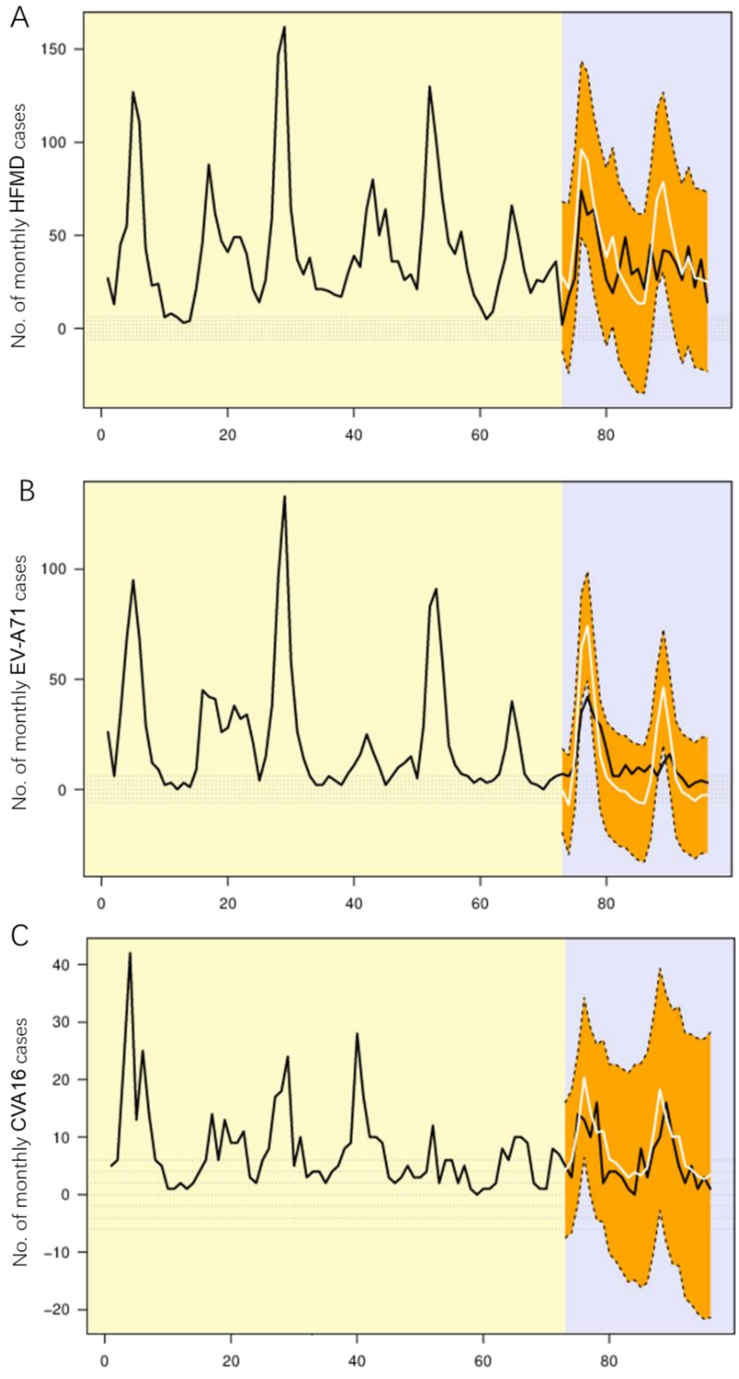

Supplementary Figure 2

EV-A71 vaccination data during 2016-2022 in Nanchang, China. (A) Number of children (<3 years) population and EV-A71vaccination rate.EV-A71 vaccination was initiated in Nanchang in June 2016. Children who received one dose of EV-A71 vaccine were included as the vaccinated population. Vaccination records of two doses were retained for each child who had received at least one dose. (B) Monthly EV-A71 vaccination in Nanchang during 2020-2022.

A

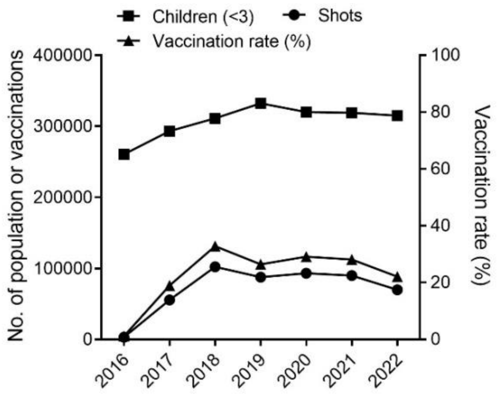

B

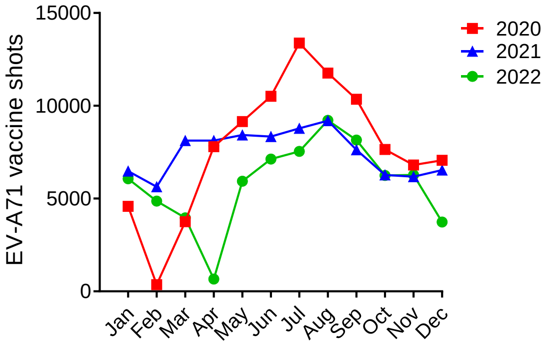

Supplement: Supplementary file 1 [file Data_Sheet_1.PDF]
